# Laboratory experiments on stranding of *Anopheles* larvae under different shoreline environmental conditions

**DOI:** 10.1186/s13071-015-0644-5

**Published:** 2015-01-21

**Authors:** Noriko Endo, Anthony E Kiszewski, Elfatih A B Eltahir

**Affiliations:** Ralph M Parsons Laboratory, Massachusetts Institute of Technology, Cambridge, USA; Natural and Applied Sciences, Bentley University, Waltham, USA

**Keywords:** Larval stranding, Larval source reduction, Water-level manipulation, Laboratory experiment

## Abstract

**Background:**

One of the concerns for future malaria epidemiology is the elevated risks of malaria around an ever-increasing number of dam sites. Controlling larval populations around reservoirs behind dams by manipulating the water levels of reservoirs could be an effective and sustainable measure for suppressing malaria epidemics; however, the effectiveness of the water-level manipulation and the contributing mechanisms have been poorly studied. In this paper, we focus on how water recession may lead to larval stranding.

**Methods:**

Larvae of *An. albimanus* were studied to assess their susceptibility to stranding under different conditions representing reservoir shoreline environments in an experimental tank (50 cm × 100 cm). The tank was initially seeded with 80 larvae uniformly, and the numbers of larvae stranded on land and remaining in water were counted (summed up to recovered larvae), following the recession of water. The vertical water drawdown rate and the proportion of stranded larvae to recovered larvae (*p*) were measured. Shoreline conditions tested were inclinations of shore slopes (2% and 4%) and surface types (smooth, vegetated, rough, ridged).

**Results:**

For the 2% slopes, the proportions of stranded larvae (*p*) increased by about 0.002, 0.004, and 0.010 as the water drawdown rate increased by a centimeter per day on the smooth, rough, and vegetated surfaces, respectively. *p* for the 4% slopes were smaller than for the 2% slopes. Unlike other surface conditions, no significant correlation between *p* and the drawdown rate was observed on the ridged surface.

**Conclusions:**

Larger proportions of *Anopheles* larvae were stranded at higher water drawdown rates, on smaller reservoir slopes, and under rough or vegetated surface conditions. Three mechanisms of larval stranding were identified: falling behind shoreline recession; entrapment in small closed water bodies; and inhabitation in shallow areas. Depending on the local vectors of *Anopheles* mosquitoes, the conditions for their favorable breeding sites correspond to the conditions for large larval stranding. If these conditions are met, water-level manipulation could be an effective measure to control malaria along shorelines of reservoirs behind dams.

## Background

Despite continuous and intensive control programs, malaria remains a major public health concern in more than one hundred countries [1]. Malaria control programs face many challenges, some of which are human-induced. Global warming associated with climate change is one of them; it may modify the extent of malaria-prone areas [2,3]. In addition, increasing dam construction to keep up with the demand for energy elevates the risk of malaria. Dams are often blamed for the creation of breeding sites for *Anopheles* mosquitoes, the vectors of malaria, associated with irrigation fields and seepage puddles downstream [4-6]. In addition, under the presence of competent vectors, reservoirs behind dams also become productive *Anopheles* breeding sites [4-6].

Currently, malaria interventions rely heavily on long-lasting insecticidal nets (LLINs) and indoor residual spraying (IRS) [7], which target adult vectors. The preference for the adult-targeting strategies has been supported by the logic of basic reproduction rates (*R*_*o*_)—the expected number of people who would be infected by a single infectious person [8-10]. Although LLINs and IRS have brought many countries significant reduction of malaria, these measures have been confronted with problems in the last two decades such as insecticide resistance, bednet utilization and durability, inadequate coverage of IRS, and lack of funding [11-16], calling into question the efficacy and the sustainability of the current malaria control strategies. On the other hand, larval source reduction, which targets aquatic-stage mosquitoes, is considered to be supplemental, as a part of Integrated Vector Management (IVM) programs, although larvae source reduction was the predominant strategy until the early twentieth century [7,17]. IVM has been touted as a more sustainable approach [7,15,18], but there are still very few practitioners. Some of the limitations of the adult-targeting strategies arise from the fact that adult mosquitoes can change their behaviors and avoid interventions [19-26]. Larvae-targeting strategies, on the other hand, are less susceptible to such problems because of the limited mobility of larvae.

**Figure 1:**
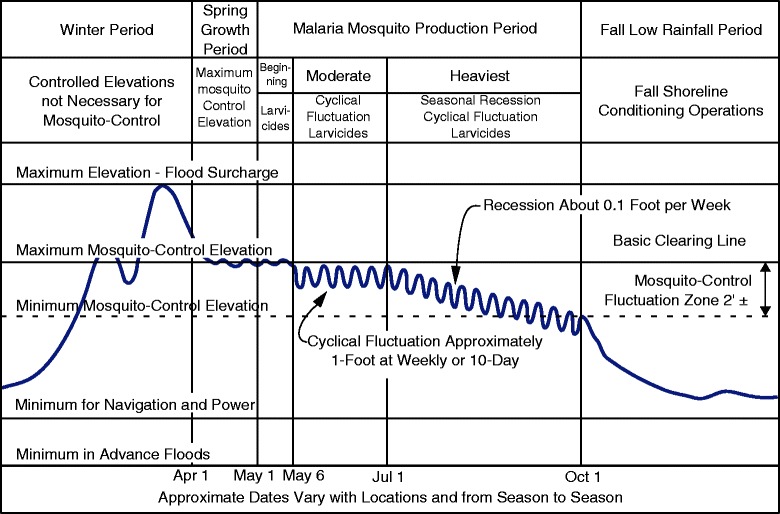
**Water-level manipulation measures applied by TVA.** The figure described the desirable phases of water-level management for mosquito control on main-river reservoirs (redrawn from [31]). During the malaria mosquito production period, cyclical fluctuations of reservoir water levels were applied at intervals of seven to ten days. In the average reservoir, it was found that a periodic fluctuation required at least one foot of vertical change to effectively reduce mosquito populations.

Larval source reduction, while less commonly selected in current malaria control programs, has been applied to seasonal pools (e.g., larviciding to kill larvae), river channels (e.g., stream-flow manipulation to flush larvae), and irrigation fields (e.g., intermittent irrigation to interrupt larval development by occasionally drying breeding sites) [5,27-30]. Around dam-related reservoirs, water-level manipulation could be used, where the reservoir water levels are controlled to create unfavorable breeding conditions for larvae. One of the most successful implementations of reservoir water-level manipulation was conducted by the Tennessee Valley Authority (TVA) in the 1930s and 1940s [31] (Figure 1), which resulted in the elimination of malaria from the US by 1947. The primary purpose of the water-level manipulation by the TVA was to eliminate vegetation and flotage from the shorelines, knowing that they provide favorable breeding sites for mosquitoes [31,32]. Another important aspect of the water-level manipulation was the direct effect on larvae; dropping water levels strand larvae to death by desiccation, and raising water levels drown larvae through increased wave actions and expose larvae to predators [31-33]. Similarly, the effects of water water-level manipulation on the control of snails, vector of schistosomiasis, were closely examined by Jobin and Michelson [34] in the lab focusing on the effect of stranding of snails, and tested successfully at some small ponds in Puerto Rico [35]. On the other hand, in spite of the successful implementation by TVA, the effects of water-level manipulation on the control of *Anopheles* larvae, have been poorly studied. The field experiment by Darrow [36] suggested that the faster drawdown rate may result in smaller number of larvae stranding, which seems to be in contradiction to Lautze’s [37] finding that the number of larvae at a shoreline was negatively correlated with drawdown rate of a reservoir water in Ethiopia. Considering the potential increase of malaria risk concurrent with dam construction, and recognizing the merits of larval source reduction, water-level manipulation to reduce larval populations deserves renewed attention.

This paper focuses specifically on the effect of water recession in shoreline environments on larval stranding. Using an experimental tank representing reservoir shoreline in a lab, we investigated (a) how larval stranding is controlled by various environmental conditions at a shoreline—water drawdown rate, reservoir slope, surface conditions and wind—and (b) what physical mechanisms contribute to stranding of larvae.

## Methods

### Experimental tank and conditions

**Figure 2:**
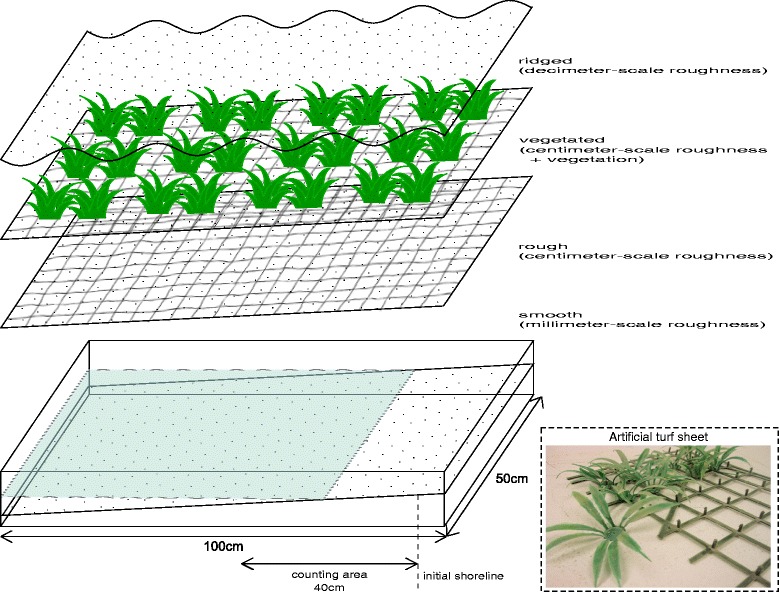
**Schematic of the experimental tank.** Surface conditions used for the slope of the experimental tank (size: about 50 cm × 100 cm) were smooth (with millimeter-scale roughness), rough (with centimeter-scale roughness), vegetated (with centimeter-scale roughness and vegetation), and ridged (decimeter-scale roughness). The vegetated surface condition was created by an artificial turf sheet shown at the bottom right. Water in the tank was reduced so that the shoreline recedes at least by 40 cm horizontally from the initial shoreline. Larvae found on the counting area (the area within 40 cm from the initial shoreline) were considered as stranded larvae.

In order to examine the stranding effect, larvae were placed in an experimental tank representing reservoir environment (Figure 2). The tank (50 cm × 100 cm) had a slope made of sand and was partially filled with distilled water. The sand slope was employed under different surface conditions to represent various reservoir environment (Figure 2). After seeding the tank with larvae, the water in the tank was drained, simulating water recession at a reservoir. Finally, the numbers of larvae stranded on the surface and those remaining in water were counted.

Table 1 summarizes the conditions tested in the experimental tank representing various reservoir shoreline environment. The variables tested were the inclination of the sand slope, larval development stage, surface type of the sand slope, and wind conditions. Tests with the same conditions except for slope were grouped together, e.g., T1 indicates the experiments with fourth instar larvae, smooth surface and no wind; it includes the experiments conducted using 2% slope (T1a) and 4% slope (T1b).

**Table 1:** **Experimental conditions**

**Test ID**	**Inclination**		**Larval stage**		**Surface type**				**Wind**			**Vertical draw down rate applied**
	**2%**	**4%**	**4th instar**	**2nd instar**	**Smooth**	**Rough**	**Vegetated**	**Ridged**	**No wind**	**Onshore wind**	**Offshore wind**	
T1a	x		x		x				x			2-54 cm/d
T1b		x	x		x				x			2-54 cm/d
T2a	x		x			x			x			2-54 cm/d
T2b		x	x			x			x			2-54 cm/d
T3a	x		x				x		x			2-54 cm/d
T3b		x	x				x		x			2-54 cm/d
T4a	x		x					x	x			2-54 cm/d
T4b		x	x					x	x			2-54 cm/d
T5	x			x		x			x			2-54 cm/d
T6	x			x			x		x			2-54 cm/d
T7	x		x			x				x		18-54 cm/d
T8	x		x			x					x	18-54 cm/d
T9	x		x				x			x		18-54 cm/d
T10	x		x				x				x	18-54 cm/d

The inclination of the sand slope used was 2% and 4%. T1-T4 were conducted with both 2% slope and 4% slope. T5-T10 were conducted using 2% slope.

The surface types tested were *smooth* (with millimeter-scale roughness horizontally and also millimeter-scale roughness vertically; hereafter “millimeter-scale roughness”), *rough* (with centimeter-scale roughness horizontally and millimeter-scale roughness vertically; hereafter “centimeter-scale roughness”), *ridged* (with decimeter-scale roughness horizontally and centimeter-scale roughness vertically; hereafter “decimeter-scale roughness”), and *vegetated* (with vegetation and centimeter-scale roughness). A *smooth* slope was made by making the sand slope even with a flat wood stick. Note that the *smooth* slope of sand is not completely flat, but granular with millimeter-scale roughness. A *vegetated* surface was introduced by inserting an artificial turf carpet (Figure 2). Approximately 60% of the area of the sand slope was covered with plastic grass. The height of the plastic grass was approximately 2 cm. In inserting the turf carpet, centimeter-scale roughness was introduced to the surface, because of both the gridded base (3 cm lattice) of the turf carpet and the inserting process itself. Hence, the *vegetated* condition included both vegetation and centimeter-scale surface roughness. In order to separate the effect of the vegetation and the centimeter-scale surface roughness, the *rough* surface condition was introduced with the same roughness condition of the surface but without vegetation. The *rough* surface condition was achieved by using the gridded base of the turf carpet only, after removing plastic grass from the base. The last surface condition tested was *ridged* slope, whose slope surface had the shape of a sinusoidal wave with a wave length of 20 cm and a wave height of 1 cm. In all cases, the surface conditions were prepared manually; thus the roughness and the shape of the sand slope varied slightly in every experiment.

The impacts of wind were examined in T7-T10. With the slope and larval stage constant (2% slope and fourth instar larvae, respectively), the effects of onshore and offshore wind were observed under the presence and the absence of vegetation. The wind was created by a small fan attached to the end of the experimental tank.

The water temperature in the tank ranged from 18 to 22°C in T1-T6, experiments without wind, where the water temperature corresponded to the air temperature in the lab. In T7-T10, experiments with wind, the water temperature dropped by a few degrees Celsius below air temperature due to evaporation cooling: it ranged from 14 to 19°C.

The water recession in the experimental tank was introduced either by a siphon or a peristaltic pump. The water levels were recorded before and after the experiment, so that the water drawdown rates could be calculated. For each test, twelve experiments were conducted with four replicates within each of the following three drawdown ranges (except for T7-T10): 2*–*6 cm/d, 6*–*18 cm/d, and 18–54 cm/d. For T7-T10, experiments were conducted only within the range of 18–54 cm/d.

The tank walls were covered with hydrophobic Teflon tape. Without Teflon tape, the hydrophilic tank material created a concave meniscus, and the surface tension from the meniscus drew larvae. To minimize this boundary effect, hydrophobic Teflon tape was applied to the tank walls, which drove away larvae by the creation of convex meniscus. As a result, less than 5% of larvae were found adhering to the walls during the experiments.

### Larvae

The STECLA strain of *An. albimanus* was used in this study. STECLA, originally isolated from collections taken from Santa Tecla, El Salvador, is maintained by NIAD/NIH as part of the BEI Resources Repository. The colony of STECLA was maintained in an incubator, whose temperature was maintained at 22°C. The larvae were fed with fish food (TetraMin) twice a day. Each experiment was conducted with 80 larvae. The larvae were initially seeded in the water in the tank uniformly. The larvae were replaced if they had been employed in three experiments or been in the tank for more than four hours. Fourth-instar larvae were employed for all sets of experiments except T5 and T6, where second-instar larvae were used.

### Larval stranding

Subsequent to water recession, larvae stranded in the area within 40 cm from the initial shoreline (hereafter referred to as the “counting area”; Figure 2) were measured as *stranded larvae*. Because larvae deposited on land can move in search of water by wriggling on damp soil, *stranded larvae* were counted only after waiting until the shoreline receded at least 3 cm horizontally from the counting area. Larvae found below the counting area were considered as *larvae remaining in water*, whether they were found on the drained area below the counting area or in the water. The number of *stranded larvae* (N_str_ ) and *larvae remaining in water* (N_rmn_ ) were counted manually. The total number of *recovered larvae* (N_rcv_ ) was calculated as N_rcv_ = N_str_ + N_rmn_. N_rcv_ was expected to be equal to the initial number of larvae seeded, i.e., |N_rcv_ − 80| = 0, but small errors were observed throughout the experiments (|N_rcv_ − 80| ≤ 3). Larvae were sometimes found dead or left uncounted hiding in water, which resulted in N_rcv_ < 80; other times more than the intended initial number of larvae were recovered (N_rcv_ > 80) due to larvae being left over from the previous experiment. The human error in counting the initial sample of 80 larvae was another cause of N_rcv_ being unequal to 80. The *proportion of stranded larvae (p) *was calculated as $p=\frac{{\mathrm{N}}_{\mathrm{str}}}{{\mathrm{N}}_{\mathrm{rcv}}}$.

## Results

### Effects of slope and surface type

**Figure 3:**
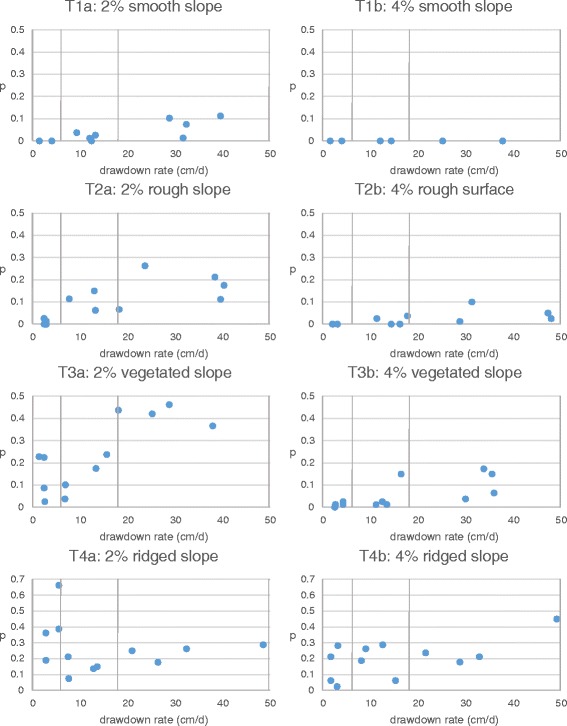
**Effects of slope and surface type on larval stranding.** Vertical drawdown rate is shown on the x-axis, and the proportion of stranded larvae (*p*) is shown on the y-axis. Three drawdown ranges (2–6, 6–18, and 18*–*54 cm/d) are shown separated by vertical lines. Twelve replicates (four replicates for each of the three drawdown range) of experiments were conducted in each test, except in T1b.

Through T1-T4, the effects of slope and surface types were tested under the three drawdown ranges (Figure 3). In T1b, no larvae were stranded after two replicates of experiments in any of the three drawdown ranges. Because additional experiments were not expected to result in any stranded larvae, no further experiments were conducted.

[Smooth slope: T1] In the tests with the smooth surface, only a small proportion of larvae were stranded. In T1a, with a 2% slope, *p* ranged only up to 0.11. *p* seemed to increase linearly with the drawdown rate. In T1b, with a 4% slope, no larvae were observed stranded (*p* = 0) at any drawdown range.

[Rough slope: T2] The 2% rough slope test (T2a) resulted in higher *p* than the 2% smooth slope test (T1a) if compared at the same drawdown range. Similarly, the 4% rough slope test (T2b) resulted in higher *p* than the 4% smooth slope test (T1b). Both T2a and T2b showed positive correlations between *p* and drawdown rate. In T2a, the minimum *p* of zero was observed at the lowest range of drawdown rate (at 2.37 cm/d and 2.84 cm/d). The highest *p* of 0.26 was observed at a drawdown rate of 23.6 cm/d (highest range), although three experiments with even higher drawdown rates of around 40 cm/d (highest range) did not result in higher *p* than that. In T2b, the minimum *p* of zero was observed in all the four experiments at the lowest range and in the two experiments at the middle drawdown range.

[Vegetated slope: T3] The vegetated slope tests with 2% slope (T3a) and 4% slope (T3b) resulted in even higher *p* than the rough slope tests, T2a and T2b, respectively, if compared at the same drawdown range. In T3a, although a certain variability in *p* (0.09 − 0.23) was found at the smallest drawdown range, the overall trend was the positive correlation between *p* and drawdown rate. The minimum *p* observed in T3a was 0.09 at a drawdown rate of 2.38 cm/d (lowest range), and the maximum *p* of 0.46 at a drawdown rate of 28.8 cm/d (highest range). The same increasing *p* trend against drawdown rate was found with the 4% vegetated slope (T3b), where the minimum *p* of zero was observed at a drawdown rate of 2.53 cm/d (lowest range), and the maximum *p* of 0.17 at a drawdown rate of 33.8 cm/d (highest range). Similar to T1 and T2, the effect of larval stranding was attenuated in the 4% slope test as compared with the 2% slope test.

[Ridged slope: T4] Unlike in T1-T3, a correlation between *p* and drawdown rate was not observed in the ridged slope tests (both T4a and T4b) (Figure 3, Table 2). *p* in T4a and T4b hovered around 0.26 and 0.21, respectively, independent of the drawdown range, with a few exceptions.

**Table 2:** **Results of linear regression analyses and correlation analyses**

**Test ID**	$\overline{\mathbf{p}}$ **(mean)**	**a (slope)**	**b (intersect)**	**R** ^**2**^	**p-value**
T1a	0.039	0.0023	−0.0022	0.60	0.0029*
T1b	0	0	0	—	—
T2a	0.23	0.010	0.098	0.60	0.0029*
T2b	0.056	0.0033	0.00034	0.48	0.011*
T3a	0.10	0.0041	0.029	0.51	0.0082*
T3b	0.021	0.0010	0.00042	0.35	0.040*
T4a	0.26	−0.0014	0.28	0.018	0.67
T4b	0.21	0.0044	0.13	0.32	0.053
T5	0.14	0.0030	0.092	0.10	0.31
T6	0.040	0.0016	0.010	0.34	0.045*

First-order linear regression analyses were applied so that $\widehat{p}$ = a × (drawdown rate) + b, where Σ($\widehat{p}$ − p)^2^ is the minimum. R^2^ and p-value are not shown for T1b because the correlation analysis is not applicable to a zero-slope line. Due to the small sample size, correlation analyses were not conducted for T7-T10.

^*^Significant at the 95% confidence interval.

### Effect of larval development stage

**Figure 4:**
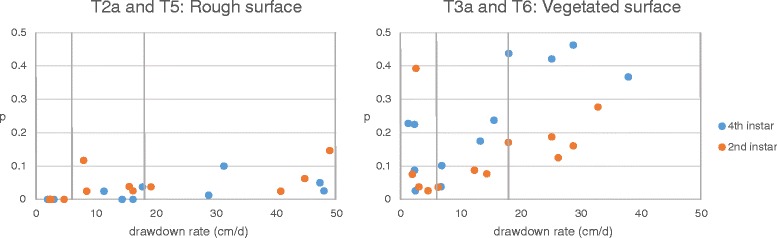
**Effect of larval development stage on larval stranding.** Vertical drawdown rate is shown in the x-axis, and the proportion of stranded larvae (*p*) is shown on the y-axis. Three drawdown ranges (2–6, 6–18, and 18–54 cm/d) are shown separated by vertical lines. Blue and orange dots represent the experiments with fourth instar and second instar larvae, respectively.

Younger larvae may have a different susceptibility to water recession than older larvae do. Two tests were carried out using second instar larvae under 2% rough and 2% vegetated surface conditions without wind (T5 and T6, respectively). The results were then compared to those from tests conducted under corresponding conditions using fourth instar larvae, i.e., T2a and T3a. (Figure 4).

[2nd and 4th instar larvae on rough slope: T5 and T2a] When the rough surface was employed with second instar larvae in T5, the trend and the magnitude of larval stranding were comparable to those in the fourth instar experiments (T2a) (Figure 4: left). Similar to the case of fourth instar larvae, second instar larvae stranded slightly more as the drawdown rate increased. The likelihood of stranding was similar for second instar larvae and for fourth instar larvae on the rough surface.

[2nd and 4th instar larvae on vegetated slope: T6 and T3a] With the vegetated slope, second instar larvae showed a smaller vulnerability to stranding than fourth instar larvae (Figure 4: right). The positive correlation between *p* and drawdown rate was observed in T6 with second instar larvae, which was consistent with T3a with fourth instar larvae.

### Effects of wind

In order to examine the impacts of wind on larval stranding, experiments were conducted using 2% rough slopes (T7 and T8) and 2% vegetated slopes (T9 and T10) with fourth instar larvae. Onshore wind was applied in T7 and T9, and offshore wind was applied in T8 and T10. When the wind was introduced, experiments conducted with small drawdown rates, which took a relatively long time, resulted in high mortality of larvae. The high larval mortality was likely because (a) larvae consumed energy by swimming or anchoring in place, resisting waves or water currents created by the wind, and (b) water temperature dropped down to 14°C due to evaporation cooling induced by wind. The energy expenditure to resist waves and currents might be an important factor to consider in controlling larvae, but the temperature drop is not relevant to the real reservoir phenomenon because the air temperature is well mixed with its surroundings and hence water temperature does not decrease easily. Because we were only interested in the effect of stranding and wanted to avoid irrelevant temperature effect, experiments for T7-T10 were conducted only within the highest drawdown range: four replications in each test. The effects causing high larval mortality were considered to be negligible within the highest drawdown range, because larvae were exposed to the experimental conditions only for a maximum of two hours. In fact, most larvae used in T7-T10 stayed healthy after experiments.

**Figure 5:**
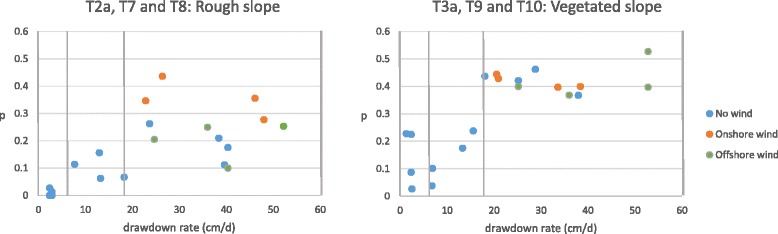
**Effects of wind on larval stranding.** Vertical drawdown rate is shown in the x-axis, and the proportion of stranded larvae (*p*) is shown on the y-axis. Three drawdown ranges (2–6, 6–18, and 18–54 cm/d) are shown separated by vertical lines. Twelve blue dots represent the experiments without wind. Orange and green dots represent the experiments with onshore wind and offshore wind, respectively.

[Wind on rough slope: T7, T8 and T2a] With the rough surface, the experiment with onshore wind (T7) resulted in a higher proportion of stranded larvae, *p*, than the no-wind test (T2a); however, the observed values of *p* in the offshore wind test (T8) were close to the values expected in T2a (Figure 5: left). Onshore wind pushed larvae to the shoreline, leaving larvae in an area which would soon be drained. The result of higher *p* with onshore wind thus sounds reasonable. The average proportion of stranded larvae in the four experiments in T7 (0.35) was approximately two times higher than the equivalent average in T2a (0.19, average of the four values of *p* obtained at the highest drawdown range). If the same mechanism worked for offshore wind, larvae would be pushed toward a deeper area; hence, only a small number of larvae would become stranded. However, the observation showed a different result. The average proportion of stranded larvae in T8 was 0.20, only a small difference from the result in T2a. In fact, in the offshore wind experiments, some larvae were observed to have been pushed to deeper water by surface waves created by the wind; however, the authors also observed that larvae tried to cling to a shallow area under offshore wind, trying not to be pushed by the waves, which could account for these surprising high values of *p*.

[Wind on vegetated slope: T9, T10 and T3a] The effect of wind on the vegetated surface is illustrated in Figure 5: right. In the onshore wind test (T9), the average proportion of stranded larvae was approximately 0.42, which was comparable to the equivalent average obtained in T3a (0.42, average of the four values of *p* obtained at the highest drawdown range). In the offshore wind test (T10), a close value of 0.42 was obtained as the average proportion of stranded larvae. Under the vegetated condition, the physical effect of wind was observed to be attenuated because the paths of wind and surface waves were partially blocked by vegetation. Consequently, the existence of wind did not significantly influence the probability of larval stranding.

## Discussion

### Mechanisms of larval stranding

**Figure 6:**
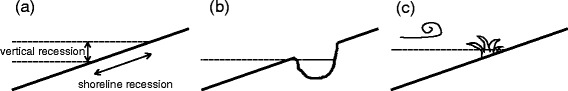
**Three mechanisms of stranding larvae. (a)** Falling behind shoreline recession: When the shoreline recession rate is high, larvae fail to overtake the receding water after deposited on land and become stranded. **(b)** Entrapment in small closed water bodies: Receding water leaves behind water and larvae at depressions. Once the water body is separated from the reservoir, larvae cannot easily leave. These separated closed water bodies may dry out soon, causing larval death. **(c)** Inhabitation in shallow areas: Larvae staying at a shallow area near the shoreline are more susceptible to stranding. Vegetation and onshore wind work to attract larvae to a shallow area.

Three mechanisms for larval stranding were identified: falling behind shoreline recession, entrapment in small closed water bodies, and inhabitation in shallow areas (Figure 6). Receding water leaves some area on a reservoir slope drained; the recession rate along the shore slope (hereafter “shoreline recession rate”) influences the extent of larval stranding. In the presence of micro-topography, water remains in small depressions during the water recession, creating closed water bodies separated from the reservoir water. Closed water bodies entrap larvae, so that larvae fail to get back into the reservoir water. Also, with the existence of vegetation or onshore wind, more larvae inhabit the shallow area near the shoreline, increasing their susceptibility to stranding. The three stranding mechanisms and their determinants are discussed further in the following sections.

#### Falling behind shoreline recession

Larvae can move on damp soil by wriggling [38,39]. Active motion of deposited larvae in search of water was observed in this experiment. After being deposited on land, larvae tried to get back into the main water body, wriggling their way. Larvae that failed to overtake the receding water finally became stranded. In this mechanism, the effect of stranding is larger if the shoreline recession rate is higher because it requires deposited larvae to move longer or faster to overtake the receding water.

Shoreline recession rates are high in the following two conditions. One is high vertical water drawdown rates. The positive correlation between *p* and drawdown rate found in this study (Table 2) is explained by this mechanism. For the tests with fourth instar larvae (T1-T4), the results of the regression analyses all showed positive slopes and relatively small intersects, except for the tests with the ridged surface (T4). Another condition is small slopes. With the same vertical drawdown rate, the shoreline recession rate is higher at a smaller slope than at a larger slope. In this study, the tests with a 2% slope experienced higher *p* than the tests with a 4% slope (Table 2), all other conditions being equal.

In addition to horizontal drawdown rates, the speed with which larvae move on the drained area is an important factor. The speed of larval wriggling is influenced by the size of sand granules, wetness of sand slope, species of *Anopheles*, etc. The development stage of larvae could also be a factor that determines their ability to wriggle. The authors expected that second instar larvae would be more susceptible to stranding than fourth instar larvae because they are less capable of wriggling their way on damp soil. However, the results did not support this expectation (Figure 4). Authors observed that second instar larvae were able to swim more easily in very shallow water, compared to fourth instar larvae. The ability to swim in shallow water makes larvae less susceptible to stranding. In this study, second instar larvae seem to have compensated for their unskillfulness in wriggling on damp soil after being deposited on land by using their ability to swim in a shallow area, in order to avoid becoming stranded.

In the case of the ridged surface (T4), receding water created relatively large and deep water bodies between ridges, where larvae seemed not to realize that the water bodies were becoming separated from the main reservoir water. By the time larvae realized that the area was becoming drained, the area was closed by ridges, leaving few paths to go back to the main reservoir water. In this situation, the rate of water recession seems not to be important.

#### Entrapment in small closed water bodies

Larvae in a closed water body can rarely find a way to move to another water body. Receding water leaves closed water bodies in depressions with various scales. For example, a rough surface creates millimeter- to centimeter-scale closed water bodies, and hoofprints become centimeter-scale closed water bodies. Even larger-scale closed water bodies can form at topographical depressions. As water recedes, larvae sometimes remain in such closed water bodies, whose water dries out after a while, leaving them with the fate of death by desiccation [33].

Tests with rough surface (T2) resulted in higher *p* than tests with smooth surface (T1), because small closed water bodies are present in T2, partially blocking larval wriggling. In the case of the ridged surface (T4), the formation of closed water bodies between the ridges made it nearly impossible for larvae to leave the area.

#### Inhabitation in shallow areas

The population susceptible to stranding is the larvae staying at shallow areas near the shoreline. Usually larvae stay in those shallow areas because wave actions and the expo- sure to predators are less there. In this study, vegetation and onshore wind were found to increase the susceptible population, lending more larvae being stranded.

**Figure 7:**
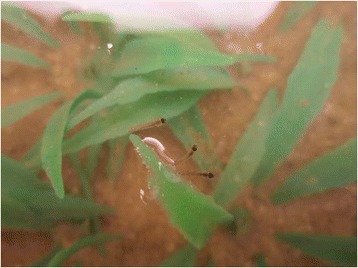
**Larvae drawn by plastic vegetation due to surface tension forces.**

Vegetated condition is favored by some species of *Anopheles* as their regular breeding sites [31,40]. In addition, larvae adhere to vegetation using surface tension [40] (Figure 7). Larvae sticking to vegetation seem to be stable and to expend less energy due to the reduced need to swim against water currents or surface waves. Larvae are drawn by vegetation with hydrophilic surfaces, which is typical for vegetation at most mosquito breeding sites. The plastic vegetation used in this study was also hydrophilic and thus drew many larvae with surface tension forces. The higher *p* observed in the vegetated slope than in the smooth slope and in the rough slope is most likely due to the mechanism of inhabitation in shallow areas due to vegetation.

The vegetated area at a reservoir is usually a narrow stretch near the shoreline especially if emergent vegetation is present. With vegetation at a shoreline, many larvae are expected to become stranded, attracted by vegetation in shallow areas, which will soon be drained if the reservoir water recedes.

The presence of wind may also influence mosquitoes to stay in a shallow area. In this study, the higher *p* was observed with onshore wind when vegetation was absent. Onshore wind creates surface waves towards the shoreline, pushing larvae to a shallower area. Offshore wind has the opposite physical effect, pushing larvae to a deeper area; however, larvae seem to realize the effect and try to cling to a shallower area in order not to be pushed away. Because of this phenomenon, this study did not find lower *p* in the offshore wind experiments as compared to no-wind experiments. The experiments were, however, conducted in less than two hours. Exposure to offshore wind for a longer time might decrease the susceptibility to stranding, but instead, larvae would face higher risk of death in reality by being pushed into deeper water. In a deep area, the effect of waves could be strong enough to drown larvae, and the risk of predation becomes high.

### Effectiveness and feasibility of water-level management at reservoirs for malaria reduction

The proportion of stranded larvae observed in this study depended on reservoir shoreline environments and water drawdown rates. The shoreline environments at which larvae are susceptible to stranding are identified as having small slopes, rough surfaces and vegetated surfaces. If *Anopheles* larvae are found around reservoirs with these conditions, water recession could effectively reduce vector population by stranding larvae. In general, small slopes of shoreline make reservoir breeding sites more attractive for *Anopheles* mosquitoes by extending shallow areas [31]. In Africa, where malaria death toll is high, *An. funestus* is one of the main vectors associated with reservoir breeding sites. *An. funestus* likes permanent water bodies, like reservoirs behind dams, with emergent vegetation [41]. Emergent vegetation contributes to the *inhabitation in shallow areas* mechanism by its nature of growing at shallow areas at the edge of reservoirs and by creating surface tension around it to anchor larvae. The coincidence of the conditions for productive *Anopheles* breeding sites and for large larval stranding suggests that deliberate manipulation of water levels can be an effective tool to control malaria around reservoirs.

The effectiveness of stranding also depends on the water drawdown rates. The highest drawdown rate tested, around 50 cm/d, may not be realistic to implement for most of reservoirs, and the effectiveness of stranding declines as drawdown rate decreases, except for ridged surface conditions. One way to achieve higher drawdown rate would be to apply periodic fluctuation of reservoir water levels, as was applied by TVA (Figure 1). At some reservoirs, shoreline surface would be close to the ridged slope condition tested in this study with the existence of microtopography. In this case, even slow drawdown could effectively strand larvae.

In this study, the maximum proportion of larval stranding rarely exceeded 0.5. This would be so because larvae were initially seeded uniformly over the tank, leaving approximately half of them outside the counting area. That is, when *p* = 0.5, nearly all the larvae became stranded when the area where they were located was drained. In reality, we may expect larvae to be concentrated in shallow areas near shoreline, making stranding more likely as water recession occurs.

Susceptibility to stranding may be species dependent. *An. albimanus* was used in this study expecting the behaviors of aquatic stage mosquitoes are not significantly different among *Anopheles* species. Although the behaviors of adult mosquitoes and preferable aquatic habitats have been well documented, little has known about the behaviors of aquatic stage mosquitoes. The effect of stranding for other *Anopheles* species is of further interest.

Stranding does not necessarily lead to larval death. Larvae can survive on damp soil, depending on their development stage, for up to 100 hours [38,39]. When a reservoir water level is kept close to the drained area, larvae may survive for a few days after being stranded because the land on which they are deposited is wet. Events such as rainfall and high waves from a reservoir supply water to stranded larvae, preventing them from dying of desiccation. Similarly, a fluctuation of reservoir water level should be carefully planned so that stranded larvae are left above water for a long enough time to die.

## Conclusions

Increasing dam construction is likely to enhance the risk of malaria throughout the world. Controlling reservoir water levels could be an effective and sustainable measure to reduce malaria around dams. Decreasing reservoir water level has an effect of stranding mosquito larvae, and hence reducing their populations. In this study, the effect of larval stranding at water recession was studied at various reservoir shoreline environments in lab settings. This paper is the first to articulate the mechanisms of larval stranding quantitatively. The paper found that larger larval stranding can be realized by higher water drawdown rates, smaller reservoir slopes, and rough or vegetated surface conditions. The fact that *Anopheles*’ favorable breeding conditions of small inclination and vegetated slope coincide with the conditions for large stranding effects would support the efficacy of water-level manipulation at reservoirs as a malaria control measure. We identified three mechanisms for larval stranding. The first mechanism, called as “falling behind shoreline recession”, occurs when larvae deposited on land fail to overtake the receding water. The second mechanism “entrapment in small closed water bodies” describes the situation where larvae are left at small puddles created at depressions behind shoreline after water recession. The third mechanism “inhabitation in shallow areas” is due to vegetation or onshore wind and keeps larvae at shallow areas near shoreline, which will soon be drained if reservoir water recedes. These findings should aid the implementation of water-level manipulation strategies at reservoirs for malaria control.
